# Changes in Cardiac Structure and Function of Recipients after Kidney Transplantation

**DOI:** 10.3390/jcm13123629

**Published:** 2024-06-20

**Authors:** Suleyman Akkaya, Umit Cakmak

**Affiliations:** 1 Department of Cardiology, Health Sciences University, Gazi Yasargil Research and Training Hospital, Diyarbakir 21070, Turkey; 2 Department of Nephrology, Health Sciences University, Gazi Yasargil Research and Training Hospital, Diyarbakir 21070, Turkey; umitccakmak@gmail.com

**Keywords:** kidney transplantation, diastolic dysfunction, echocardiography, pulmonary artery pressure

## Abstract

**Background:** Chronic kidney disease (CKD) elevates the risk of cardiovascular disease (CVD) and mortality. Uremic cardiomyopathy, frequently observed in CKD and end-stage renal disease (ESRD), involves alterations in cardiac structure and function, which may reverse post-kidney transplantation, although data remain controversial. This study examines the relationship between graft function and changes in cardiac parameters pre- and post-transplantation in kidney transplant recipients. **Methods:** A total of 145 pediatric and adult recipients of living or deceased donor kidney transplants were enrolled at Gazi Yaşargil Training and Research Hospital. This cohort study utilized transthoracic echocardiographic (TTE) imaging pre-transplant and at least two years post-transplant. Echocardiographic parameters were analyzed using standard techniques. **Results:** The mean age of the participants was 35 years, with 60% male. The average dialysis duration prior to transplantation was 27 months. Most recipients (83.4%) received kidneys from living donors. Left ventricular diastolic dysfunction increased significantly post-transplant (*p* < 0.05), while other cardiac dimensions and functions, such as ejection fraction and pulmonary artery pressure, showed no significant change (*p* > 0.05). Notably, diastolic dysfunction worsened in patients with dysfunctional grafts (GFR < 45), correlating with increased pulmonary artery pressure post-transplant. The rate of antihypertensive drug use and the prevalence of diabetes mellitus increased significantly post-transplant (*p* < 0.05). **Conclusions:** This study demonstrates that left ventricular diastolic dysfunction present before kidney transplantation continues to persist post-transplantation in patients with end-stage renal disease undergoing chronic kidney disease treatment. Furthermore, it shows an increased rate of pulmonary artery pressure and pericardial effusion in patients with dysfunctional grafts after transplantation. Further research is required to explore strategies to reverse uremic cardiomyopathy and reduce cardiovascular risk in these patients.

## 1. Introduction

Chronic kidney disease (CKD) is a strong risk factor for cardiovascular disease (CVD) and mortality [1]. The presence of CVD risk factors leads to the progression of existing CVD and increased mortality in CKD patients. In patients with advanced CKD and end-stage renal disease (ESRD), kidney transplantation treatment is known to improve survival more than dialysis treatment [2]. The heart and kidneys are interconnected organs. When the function of one of these organs is impaired, the hemodynamic, neurohumoral, immunologic, and biochemical feedback pathways of the other may be affected, initiating organ damage [3]. This condition is called cardiorenal syndrome (CRS) and has become a common occurrence due to the unfavorable clinical outcome of heart and kidney disease. Patients who develop CRS have a high risk of hospitalization and mortality; when renal and heart failure coexist, progressive dysfunction of each organ occurs, and survival is adversely affected [4].

Uremic cardiomyopathy is frequently observed in CKD, including ESRD [5]. Uremic cardiomyopathy is defined as changes in cardiac structure and function caused by the uremic state. The magnitude and characteristics of changes in cardiac structure and function and their relationship to allograft dysfunction affecting kidney transplant recipients are not fully understood. Among the cardiac changes occurring in these patients, left ventricular hypertrophy is the most commonly reported change [6]. In addition to these changes, changes in right and left ventricular and atrial volume, systolic and diastolic function, pulmonary artery pressure, and the rate of pericardial effusion have been reported. Kidney transplantation offers a significant survival benefit over dialysis for patients with ESRD, improving both quality of life and cardiovascular outcomes. The prevalence of cardiovascular complications remains high after kidney transplantation, and cardiovascular events account for 36–55% of the causes of death in kidney transplant recipients [7]. Numerous factors that could lead to uremic cardiomyopathy are improved by the restoration of renal function linked with KT. It is nevertheless somewhat debatable whether kidney transplantation improves diastolic and systolic function and lowers left ventricular mass index (LVMI) and volumes [8]. However, the reversal of cardiac abnormalities post-transplantation is variable and remains a subject of ongoing research [8]. The complexity of cardiac disease in kidney transplant recipients stems from a variety of factors, including pre-existing cardiomyopathy, the surgical and recovery processes associated with transplantation, and the effects of necessary post-operative medications, particularly immunosuppressants [9]. Factors associated with cardiac remodeling after long-term renal transplantation have not been adequately reported. This study aims to delineate the relationship between graft function and the progression or regression of cardiac parameters in recipients before and after kidney transplantation. By understanding these dynamics, we can better strategize interventions to optimize cardiac health in kidney transplant recipients, potentially enhancing both graft and patient survival rates.

## 2. Materials and Methods

### 2.1. Study Population

This study included 145 pediatric and adult kidney transplant recipients who underwent living or deceased donor kidney transplantation at Gazi Yasargil Training and Research Hospital. This study was designed to compare transthoracic echocardiographic (TTE) imaging data before kidney transplantation with TTE data at least 2 years after kidney transplantation. This study was approved by the Scientific Research Ethics Committee of Diyarbakır Provincial Health Directorate Gazi Yaşargil Training and Research Hospital with the decision dated 29 September 2023 and numbered 524. Patients who underwent heart transplantation and had no TTE imaging or died during follow-up were excluded. The baseline TTE data of all patients before the kidney transplant operation and the TTE data performed during follow-up 2 years after the kidney transplant operation were recorded. Finally, a total of 145 patients were analyzed. All patients were receiving immunosuppressive drugs based on the standard protocol, including cyclosporine, tacrolimus, mycophenolate mofetil, and prednisone.

### 2.2. Transthoracic Echocardiography

Echocardiograms were performed at the Gazi Yaşargil Training and Research Hospital by experienced cardiologists according to current recommendations using a Vivid E95 echocardiographic system (GE Healthcare, Tokyo, Japan) and a 2.5 MHz transducer and were all reviewed offline with dedicated software. Comprehensive measurements of conventional functional and structural echocardiographic parameters for both ventricles were performed in line with cardiac chamber quantification standards. The following diastolic parameters were recorded: maximal early and late mitral inflow velocities with pulsed-wave Doppler (PW) (E wave, A wave, A wave duration, E wave deceleration time, and E/A ratio), lateral and septal mitral annular e’ velocity with TDI, average E/e’ ratio, peak velocity of tricuspid regurgitation jet by continuous-wave Doppler (CW), and LA biplane volume index. Patients had normal or preserved systolic function, no (or grade 1) diastolic dysfunction, no significant valvular disease, mild concentric hypertrophy, and mild left atrial dilatation. The two-dimensional transthoracic echocardiography also measured the ascending aorta diameter (Aod), left atrial diameter (LAD), left ventricular end-diastolic diameter (LVDd), left ventricular end-systolic diameter (LVDs), interventricular septal thickness (IVS), posterior left ventricular wall thickness (LVPW), left ventricular ejection fraction (LVEF), and velocities of mitral valve diastolic flow (E1, A1), as well as mitral ring lateral wall and septal motion velocities (E’).

### 2.3. The Data

Age, gender, body mass index (BMI), dialysis type (pre-emptive, hemodialysis, peritoneal dialysis), dialysis duration, transplant type (living, deceased donor), CKD etiology, graft loss and its cause, immunosuppressive drugs (cyclosporine, tacrolimus, prednisolone, everolimus, mycophenolate mofetil), antihypertensive drugs (ACEi, ARBs, betablockers, calcium channel blockers, diuretics), and the development of HT and DM before and after transplantation were recorded. History of coronary artery disease, coronary angiography results, myocardial scintigraphy results, or the presence of previous acute coronary syndrome before and after transplantation were determined and recorded. Cardiac parameters were recorded and compared before and after transplantation by TTE imaging. The GFR measurements and TTE measurements were performed in the same period before and after transplantation. The same equipment and standardized protocols were used for all echocardiographic assessments. GFR was estimated using the Chronic Kidney Disease Epidemiology Collaboration equation (CKD-EPI) equation for adults, and the Schwartz equation was used for pediatric patients [10].

### 2.4. Statistical Analyses

Mean, standard deviation, median minimum and maximum, frequency, and ratio values were used in descriptive statistics of the data. The distribution of variables was measured by the Kolmogorov–Smirnov and Shapiro–Wilk tests. An independent sample *t*-test and the Mann–Whitney u-test were used to analyze quantitative independent data. Wilcoxon’s test was used to analyze dependent quantitative data. A Chi-square test was used to analyze qualitative independent data, and Fisher’s test was used when the chi-square test conditions were not met. McNemar’s test was used to analyze qualitative dependent data. SPSS, version 25.0 (IBM Inc., Armonk, NY, USA) program was used in the analyses.

## 3. Results

### 3.1. Demographic and Clinical Outcomes

This study included 180 kidney transplant recipients. Eight patients were excluded from the study due to graft loss before the two-year follow-up. A total of 12 patients were excluded because of a second transplantation. Five patients were excluded because of exitus. In total, 10 patients were excluded because of missing post-transplant TTE and data. The mean age of 145 kidney transplant patients who were included in study was 35 years, and 60% of these patients were male. A total of 55.2% of the patients were receiving hemodialysis (HD), 39.3% were receiving pre-emptive, and 5.5% were receiving continuous outpatient peritoneal dialysis. The mean duration of the dialysis treatment before transplantation was 27 months (27.3± 44.7). Among the patients who received kidney transplantation, 83.4% received a kidney from living donors, and 14.5% received a kidney from cadaver. The demographic and clinical results are given in Table 1.

### 3.2. Changes in TTE Imaging before and after Kidney Transplantation

**Figure 1:**
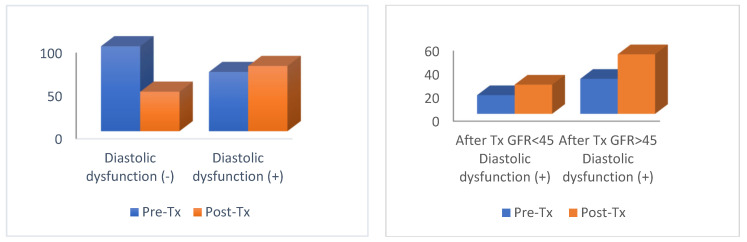
Trends in diastolic dysfunction pre- and post-kidney transplantation. This bar chart depicts the percentage of patients with and without diastolic dysfunction before and after kidney transplantation (**left** graph) and impact of GFR on diastolic dysfunction pre- and post-transplantation. The graph illustrates the prevalence of diastolic dysfunction among patients with different levels of GFR before and after kidney transplantation (**right** graph).

Left ventricular interventricular septum measurements of the heart after kidney transplantation did not show a significant (*p* > 0.05) change compared those to before kidney transplantation. The proportion of patients with left ventricular diastolic dysfunction increased significantly (*p* < 0.05) after kidney transplantation compared to that before kidney transplantation (Table 2) (Figure 1). The posterior left ventricle, right atrium (RA), left atrium (LA), pulmonary artery pressure (PAB max), and ejection fraction (EF) values after renal transplantation did not change significantly (*p* > 0.05) compared to those before renal transplantation. The right ventricle (RV) value increased significantly (*p* < 0.05) after renal transplantation compared to that before renal transplantation. The rate of LVH, mitral insufficiency, aortic insufficiency, and pericardial effusion did not change significantly (*p* > 0.05) after kidney transplantation compared to before kidney transplantation. The rate of HT, antihypertensive drug use, and DM increased significantly (*p* < 0.05). The rate of coronary artery disease (CAD) and acute coronary syndrome (ACS) after kidney transplantation did not change significantly (*p* > 0.05) compared to before kidney transplantation (Table 2).

The age and gender distribution of the patients did not differ significantly (*p* > 0.05) between the GFR < 45 and GFR > 45 groups after kidney transplantation. The height, weight, and BMI values did not differ significantly (*p* > 0.05) between the GFR < 45 and GFR > 45 groups (Table 3). Dialysis type and duration of dialysis did not differ significantly (*p* > 0.05) between the GFR < 45 and GFR > 45 groups. Donor proximity did not differ significantly (*p* > 0.05) between the GFR < 45 and GFR > 45 groups. The deceased donor transplantation rate was significantly (*p* < 0.05) higher in the GFR < 45 group than in the GFR > 45 group (Table 3).

There was no significant (*p* > 0.05) difference in the rate of left ventricular diastolic dysfunction between the GFR < 45 and GFR > 45 groups before and after kidney transplantation (Figure 1). In the GFR < 45 group, the rate of left ventricular diastolic dysfunction increased significantly (*p* < 0.05) after kidney transplantation compared to that before kidney transplantation. In the group with GFR > 45, the rate of diastolic dysfunction increased significantly (*p* < 0.05) after kidney transplantation compared to that before kidney transplantation (Table 4). The RV value before and after kidney transplantation did not differ significantly (*p* > 0.05) between the groups with GFR < 45 and GFR > 45. In the GFR < 45 group, the RV value after kidney transplantation did not change significantly (*p* > 0.05) compared to that before kidney transplantation. In the group with GFR > 45, the RV value increased significantly (*p* < 0.05) after kidney transplantation compared to that before kidney transplantation. There was no significant (*p* > 0.05) difference between the GFR < 45 and GFR > 45 groups in terms of RV increase before/after kidney transplantation (Table 5).

**Figure 2:**
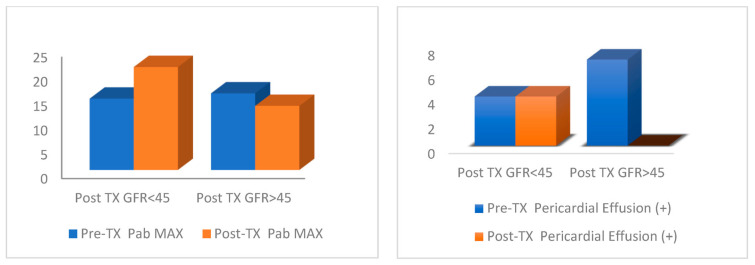
Comparison of maximum pulmonary artery pressure (PAB Max) before and after kidney transplantation (TX) (**left** graph) and pericardial effusion rates before and after kidney transplantation. The chart details the percentage of patients experiencing pericardial effusion, categorized by GFR (**right** graph).

The PAB max value did not differ significantly (*p* > 0.05) between the GFR < 45 and GFR > 45 groups before kidney transplantation compared to after kidney transplantation. The PAB max value after kidney transplantation in the GFR < 45 group was significantly (*p* < 0.05) higher than that in the GFR > 45 group (Figure 2). In the group with GFR < 45, the PAB max value after kidney transplantation increased significantly (*p* < 0.05) compared to that before kidney transplantation. In the group with GFR > 45, the PAB max value after TX decreased significantly (*p* < 0.05) compared to that before kidney transplantation. In the group with GFR < 45, the change in PAB max before/after kidney transplantation was significantly (*p* < 0.05) higher than in the group with GFR > 45. An increase was observed in the group with GFR < 45, and a decrease was observed in the group with GFR > 45 (Table 5). In the group with GFR < 45, the EF value before and after kidney transplantation was significantly (*p* < 0.05) lower than in the group with GFR > 45. In the GFR < 45 group, the EF value after kidney transplantation did not change significantly (*p* > 0.05) compared to that before kidney transplantation. In the GFR > 45 group, the EF value after kidney transplantation did not change significantly (*p* > 0.05) compared to that before kidney transplantation. There was no significant (*p* > 0.05) difference between the groups with GFR < 45 and GFR > 45 in terms of EF change before/after kidney transplantation (Table 5). 

There was no significant (*p* > 0.05) difference in LVH rate between the GFR < 45 and GFR > 45 groups before and after kidney transplantation. In the group with GFR < 45, the LVH rate after kidney transplantation did not change significantly (*p* > 0.05) compared to that before kidney transplantation. In the group with GFR > 45, the LVH rate after kidney transplantation did not show a significant (*p* > 0.05) change compared to that before kidney transplantation (Table 6).

The pericardial effusion rate before kidney transplantation did not differ significantly (*p* > 0.05) between the GFR < 45 and GFR > 45 groups. The rate of pericardial effusion after TX was significantly (*p* < 0.05) higher in the GFR < 45 group than in the GFR > 45 group. In the group with GFR < 45, the rate of pericardial effusion after kidney transplantation did not change significantly (*p* > 0.05) compared to that before TX. In the group with GFR > 45, the rate of pericardial effusion after TX decreased significantly (*p* < 0.05) compared to that before TX (Table 7).

There was no significant (*p* > 0.05) difference between the groups with GFR < 45 and GFR > 45 before and after kidney transplantation. In the group with GFR < 45, the HT rate after kidney transplantation increased significantly (*p* < 0.05) compared to that before kidney transplantation. In the group with GFR > 45, the rate of HT after kidney transplantation increased significantly (*p* < 0.05) compared to that before kidney transplantation. The rate of antihypertensive drug use before and after kidney transplantation did not differ significantly (*p* > 0.05) between the groups with GFR < 45 and GFR > 45. In the group with GFR < 45, the rate of antihypertensive drug use after kidney transplantation increased significantly (*p* < 0.05) compared to that before kidney transplantation. In the group with GFR > 45, the rate of antihypertensive drug use after kidney transplantation increased significantly (*p* < 0.05) compared to that before kidney transplantation. The rate of DM before and after kidney transplantation did not differ significantly (*p* > 0.05) between the groups with GFR < 45 and GFR > 45. In the group with GFR < 45, the DM rate after kidney transplantation did not change significantly (*p* > 0.05) compared to that before Kidney transplantation. In the group with GFR > 45, the DM rate after kidney transplantation increased significantly (*p* < 0.05) compared to that before kidney transplantation. The EX rate was significantly (*p* < 0.05) higher in the group with GFR < 45 than in the group with GFR > 45.

The posterior measurement was larger in non-pre-emptive KTx patients before kidney transplantation (1.08 vs. 0.98, *p* < 0.001) (Table 8). There was a difference between posterior measurement before and after kidney transplantation in pre-emptive patients (1.03 vs. 0.98, *p* = 0.049). The change in RA measurement before and after kidney transplantation was greater in pre-emptive patients (−0.07 vs. −0.008, *p* = 0.045). There was a difference between pre- and post-kidney transplantation measurements in pre-emptive patients (3.33 vs. 3.2, *p* = 0.026). There was a difference in the LA measurement between pre-emptive and non-pre-emptive KTx patients (3.68 vs. 3.47, *p* = 0.041). Pericardial effusion was more common in pre-emptive patients before kidney transplantation (11.4% vs. 1.8%, *p* = 0.033). There was a significant decrease in the rate of pericardial effusion in patients receiving non-pre-emptive KTx before and after kidney transplantation (11.4% vs. 2.3%, *p* = 0.039). LVH was more common in pre-emptive patients before kidney transplantation (66.7% vs. 43.2%, *p* = 0.006) (Table 9).

There was a difference between post-kidney transplant RV and pre-kidney transplant RV in pre-emptive patients (3.25 vs. 2.98, *p* < 0.001). Non-pre-emptive KTx recipients had a higher proportion of patients with diastolic dysfunction before kidney transplantation compared to pre-emptive patients (39.8% vs. 19.3%, *p* = 0.010). The rate of diastolic dysfunction in pre-emptive patients was higher after kidney transplantation than before kidney transplantation (45.6% vs. 19.3%, *p* = 0.001). In patients receiving non-pre-emptive KTx, the rate of diastolic dysfunction was higher after kidney transplantation than before kidney transplantation (56.8% vs. 39.8%, *p* = 0.014) (Table 10).

## 4. Discussion

Dialysis treatment in ESRD is an important risk factor for CVD. Early transplantation and short duration of time on dialysis have been associated with good survival [11]. Uremic and inflammatory processes, fluid overload, or hypotension during dialysis treatment are important etiologic factors that can be counted in terms of CVD risk for chronic kidney disease patients. In particular, hypervolemic state and intradialytic hypotension during hemodialysis treatment have been shown to be associated with mortality in adults [12]. Intradialytic hypotension, hypervolemic, hypervolemic, hyperuremic state, and inflammatory processes occurring in patients undergoing hemodialysis can cause chronic cardiac pathological conditions by causing decreased coronary flow and myocardial damage in the heart [13]. Left ventricular diastolic function, one of the cardiac pathologies, can be impaired both in dialysis patients and in kidney recipients after transplantation [14,15,16,17]. Some studies have demonstrated a relationship between the length of dialysis time before kidney transplantation and left ventricular stiffness [18]. Left ventricular diastolic dysfunction detected while the patient is on dialysis may persist after renal transplantation [15,16,19]. Hypervolemia and hyperparathyroidism may cause left ventricular diastolic dysfunction [14,20]. Long-term dialysis treatment prior to kidney transplantation may cause irreversible myocardial structural and metabolic changes [21]. The presence of persistent systemic hypertension, calcineurin inhibitor therapy [22], and sympathetic hyperactivity may lead to cardiac structural and functional defects that persist even after successful kidney transplantation [23,24]. Several studies have demonstrated changes in left ventricular structure and function in end-stage renal failure and subsequent renal transplantation [25,26,27]. A controlled study by De Lima et al. [28] reported a small decrease in the E/A ratio at 1-year follow-up, while Deng et al. [29] reported a small increase. An et al. reported that recipients with moderate diastolic dysfunction before transplantation showed a significant decrease in the E/A ratio at 12 months, while those with mild dysfunction showed a significant change only at 5-year follow-up [30]. In our study, the left ventricular diastolic dysfunction rate was found to be significantly higher after kidney transplantation when compared to before and after kidney transplantation. At the same time, the rate of left ventricular diastolic dysfunction increased significantly after kidney transplantation compared to that before kidney transplantation between the two groups with functional graft and dysfunctional graft. The reversal of uremic cardiomyopathy caused by the uremic state of the body before kidney transplantation plays a key role in reducing cardiovascular morbidity and mortality in ESRD. Although no targeted therapy has been shown to achieve this, it is generally assumed that the improvement in renal function with kidney transplantation reverses the observed cardiac changes. The majority of uncontrolled echocardiographic studies have reported significant reductions in left ventricular mass index (LVMI); however, TTE is unreliable in measuring LVMI as it may be inaccurate in situations where large volume fluctuations occur. Cardiovascular magnetic resonance imaging is more accurate and reproducible and is considered the gold standard imaging modality for patients with ESRD [5]. Following renal transplantation, many traditional risk factors for cardiovascular disease persist, and in some cases, they may re-develop. Steroids and calcineurin inhibitors used in treatment after kidney transplantation are known to cause complications such as hypertension, dyslipidemia, and diabetes. There are also non-traditional risk factors such as uremia, proteinuria, and chronic inflammation [31]. The excessive inflammatory process induced by the treatment regimens used after kidney transplantation may perpetuate cardiac structural abnormalities previously induced by dialysis. We also observed that the cardiac diastolic dysfunction caused by the physiopathologic condition that occurred during dialysis treatment in patients with ESRD before kidney transplantation did not change with transplantation treatment. We observed that the diastolic dysfunction that occurred in patients before transplantation did not improve after transplantation and even increased significantly. In our study, we observed that the physiopathological changes in the heart caused by the uremic status and other markers that existed before transplantation did not change, and the pathological conditions in the heart even increased.

Hemodialysis is a renal replacement therapy option that can improve the clinical outcomes of end-stage renal failure and reduce various complications, but we do not have sufficient information to compare the effects of hemodialysis on myocardial structure between patients undergoing hemodialysis treatment before kidney transplantation and those undergoing pre-emptive kidney transplantation. Therefore, in this study, we aimed to investigate whether the treatment strategy based on pre-emptive kidney transplantation improves cardiac structure and function after transplantation compared to the approach involving chronic dialysis before transplantation. In our study, 39.3% of 145 patients underwent pre-emptive kidney transplantation, while 59.7% underwent kidney transplantation while on dialysis. Pre-transplant diastolic dysfunction was significantly higher in the chronic dialysis group than in the pre-emptive group, but diastolic dysfunction did not improve in either group after kidney transplantation, and it even increased significantly. The reason for the significant increase in diastolic dysfunction rates after transplantation compared to pre-transplantation in patients is the continuation of the negative situation created by the uremic and other chronic markers in the heart before transplantation. In addition, immunosuppressive and steroid drugs used in transplantation treatment after transplantation may contribute to the increase in vascular and cellular fibrosis in the myocardium due to the effect of changes in the inflammation cascade, leading to an increase in the rate of diastolic dysfunction. As a result, fibrosis that develops and continues to increase at the microvascular level in the myocardium causes an increase in the rate of diastolic dysfunction by creating a contraction defect in the myocardium. For this reason, the transplantation treatment of patients with chronic renal failure should be performed as soon as possible to shorten the time spent on dialysis and to reduce some irreversible pathologic conditions occurring in cardiac structures. Highly powered and controlled studies are needed to reverse uremic cardiomyopathy and ameliorate the increased cardiovascular risk associated with ESRD. Arterial hypertension is common in most ESRD cases before transplantation treatment [32]. However, specific immunosuppressive regimens used after kidney transplantation, donor age, and post-transplant graft dysfunction contribute to the persistence of post-transplant hypertension [33]. The pathogenesis of arterial hypertension in kidney transplant recipients is complex. Calcineurin inhibitors (CNIs), which are frequently used after transplantation, may increase peripheral vascular resistance by causing arteriolar vasoconstriction, decrease the glomerular filtration rate, activate the renin–angiotensin system, and inhibit atrial natriuretic peptide, ultimately leading to an increase in extracellular volume [34]. Glucocorticoids may also impair the urinary excretion of water and salt. In addition, the worsening of graft function may lead to an increase in extracellular volume and inappropriate renin production, leading to hypertension [35]. High blood pressure may lead to the worsening of diastolic dysfunction and impaired graft function. In our study, the significant increase in post-transplant hypertension in our kidney transplant recipients can be explained as the most important reason for the worsening of diastolic dysfunction after transplantation for the reasons mentioned above. Therefore, the modification of current immunosuppression therapy and control of blood pressure have an important role in reducing post-transplant cardiovascular complications.

In our study, our patient group fits into the type 4 CRS patient group as we looked at the effect of chronic kidney disease on cardiac structure and function, and this is associated with cardiac ventricular hypertrophy, diastolic dysfunction, and cardiovascular events. Uremia in end-stage renal disease patients is characterized by the accumulation of uremic toxins and inflammatory cytokines in the blood, leading to inflammation, oxidative stress, endothelial dysfunction, and the consequent acceleration of atherosclerosis and progression of CKD. Furthermore, increased neurohumoral activation in these patients results in cardiac remodeling, left ventricular hypertrophy, vascular calcification, ischemia, coronary artery disease, and heart failure [36]. Cardiac clinical conditions seen in patients with CKD include decreased ejection fraction, increased end-systolic and end-diastolic left ventricular diameters and volumes, endocardial and epicardial fibrosis, left ventricular hypertrophy and left ventricular dilatation, and left ventricular systolic dysfunction. Cardiovascular morbidity and mortality are higher in patients with ESRD than in the general population [37].

Pulmonary hypertension (PH) has been associated with a significantly increased risk of overall mortality and cardiovascular mortality in patients with ESRD [38]. PH is also common in renal transplant recipients, associated with worsening 5-year transplant outcomes and the increased incidence of graft dysfunction [39]. In studies on PH in renal transplant recipients by Issa et al. [40] and Wang et al. [41], the relationship between the pre-transplant TTE findings of PH and post-transplant graft dysfunction and patient outcomes has been shown. In the Issa study, 215 patients were examined, and after 23 months of follow-up, a higher risk of mortality was observed in patients with PH left undiagnosed on TTE [39]. Wang et al. showed that high pulmonary artery pressure before transplantation did not increase the 4-year mortality rate or graft loss. In the same study, an association between high pulmonary artery pressure and decreased graft function, which manifested itself in lower eGFR at the 2-year follow-up of patients after transplantation, was found [41].

In our study, no significant change in pulmonary artery pressure was observed in pre- and post-transplant TTE, whereas post-transplant pulmonary artery pressure was significantly higher in the group with graft dysfunction (GFR < 45) compared to pre-transplant. In the group with normal graft function (GFR > 45), a significant decrease in post-transplant pulmonary artery pressure was observed. In addition, in our study, the rate of pericardial effusion was significantly higher in the group with GFR < 45 after TX than in the group with GFR > 45 (*p* < 0.05). In addition, the rate of patients with pericardial effusion after TX in the group with GFR > 45 decreased significantly compared to that before TX (*p* < 0.05). We can evaluate the result of our study as follows: after kidney transplantation, depending on the condition of the graft, a different physiopathologic situation will occur depending on whether the kidney is functioning or not. If there is graft dysfunction after transplantation, a hypervolemic state occurs due to the loss of the filtration ability of the kidney. In addition, the potential deleterious effects of increased afterload, sustained volume overload, and persistent overactivity of the renin–angiotensin–aldosterone and sympathetic nervous systems will cause a load on the right and left ventricle of the heart, leading to increased pulmonary artery pressure. However, after transplantation, pathophysiologic events such as endothelial dysfunction, metabolic and neurohumoral changes, anemia, and vascular calcifications will also cause an increase in pulmonary artery pressure, in addition to the formation of a hypervolemic state with impaired renal function. Due to the mechanism of increased pulmonary artery pressure, patients have experienced an increased rate of pericardial effusion as a sign of overload. This situation showed that follow-up after transplantation is very important.

## 5. Conclusions

This study shows that left ventricular diastolic dysfunction in the heart before kidney transplantation persists after kidney transplantation in patients with end-stage renal failure who are followed up with chronic kidney disease treatment. This study also shows that the rate of pulmonary artery pressure and pericardial effusion is increased in patients with dysfunctional grafts after kidney transplantation. Rapid referral of patients with end-stage renal failure to renal transplantation therapy will prevent the occurrence of diastolic dysfunction in the heart caused by the pre-existing uremic state before renal transplantation. Maximal treatment regimens after renal transplantation will prevent the graft from becoming dysfunctional and reduce the increase in pulmonary artery pressure and pericardial effusion in the heart.

## Figures and Tables

**Table 1:** Demographic and clinical characteristics of kidney transplant recipients.

		Min–Max			Median Mean ± sd/n%			
Age		11.0	–	65.0	34.0	35.2	±	12.7
Gender	Male					87		60%
	Female					58		40%
Length		140.0	–	194.0	165.0	164.1	±	9.8
Weight		27.0	–	95.0	63.0	62.8	±	14.9
BMI		13.7	–	36.7	22.3	23.2	±	4.7
Dialysis Type	Pre-emptive					57		39.3%
	HD					80		55.2%
	CAPD					8		5.5%
Transplant Type	Deceased					21		14.5%
	Live					124		85.5%
CRF Etiology	Unknown Causes					79		54.5%
	HT					28		19.3%
	Glomerulonephritis					12		8.3%
	Nephrolithiasis					9		6.2%
	DM					5		3.4%
	Neurogenic Bladder					3		2.1%
	VUR					3		2.1%
	PKBH					2		1.4%
	Congenital Hypoplastic Kidney					1		0.7%
	Tubulointerstitial Nephritis					1		0.7%
	Pre-eclampsia					1		0.7%
	FMF-Amyloidosis					1		0.7%

Abbreviations: HD: hemodialysis, CAPD: continuous ambulatory peritoneal diuresis, CRF: chronic renal failure, HT: hypertension, DM: diabetes mellitus, VUR: vesicoureteral reflux, FMF: familial Mediterranean fever.

**Table 2:** Comparative analysis of transthoracic echocardiographic (TTE) measurements before and after kidney transplantation.

		Pre-TX			Post-TX			*p*	
		Median	Mean ± sd/n%		Median	Mean ± sd/n%			
TTE									
LVH	(−)		76	52.4%		73	50.3%	0.780	^N^
	(+)		69	47.6%		72	49.7%		
Mitral Regurgitation	(−)		80	55.2%		87	60.0%	0.427	^N^
	Mild level		55	37.9%		44	30.3%		
	Medium level		9	6.2%		13	9.0%		
	Advanced level		1	0.7%		1	0.7%		
Atrial Regurgitation	(−)		117	80.7%		120	82.8%	0.678	^N^
	Mild level		26	17.9%		20	13.8%		
	Medium level		2	1.4%		4	2.8%		
	Advanced level		0	0.0%		1	0.7%		
Pericardial Effusion	(−)		134	92.4%		141	97.2%	0.118	^N^
	(+)		11	7.6%		4	2.8%		
HT	(−)		105	72.4%		38	26.2%	0.000	^N^
	(+)		40	27.6%		107	73.8%		
Antihypertensive Drug Use	(−)		107	73.8%		35	24.1%	0.000	^N^
	(+)		38	26.2%		110	75.9%		
DM	(−)		140	96.6%		119	82.1%	0.000	^N^
	(+)		5	3.4%		26	17.9%		
CAD	(−)		138	95.2%		132	91.0%	0.070	^N^
	(+)		7	4.8%		13	9.0%		
ACS	(−)		141	97.2%		142	97.9%	1.000	^N^
	(+)		4	2.8%		3	2.1%		

^N^ McNemar’s test.

**Table 3:** Comparative analysis of kidney transplant recipients by GFR.

		GFR < 45 (n = 42)				GFR > 45 (n = 103)				*p*	
		Mean ± sd/n%			Median	Mean ± sd/n%			Median		
Age		34.9	±	13.8	35.0	35.3	±	12.4	34.0	0.859	^t^
Gender	Male	24		58.5%		62		61.4%		0.753	^X^2^^
	Female	17		41.5%		39		38.6%			
Length		163.7	±	9.7	165.0	164.3	±	9.9	164.0	0.718	^t^
Weight		61.0	±	13.2	60.5	63.5	±	15.5	64.5	0.375	^t^
BMI		22.9	±	4.7	21.7	23.3	±	4.7	22.3	0.569	^m^
Dialysis Type	Pre-emptive	15		35.7%		42		40.8%		0.159	^X^2^^
	HD	27		64.3%		53		51.5%		0.571	^X^2^^
	CAPD	0		0.0%		7		6.8%		1.000	^X^2^^
	HD + CAPD	0		0.0%		1		1.0%		0.083	^X^2^^
Dialysis Duration (Month)		33.0	±	50.0	8.5	25.0	±	42.4	2.0	0.215	^m^
Transplant Type	Deceased	12		30.0%		9		8.57%		0.001	^X^2^^
	Live	28		70.0%		96		91.42%			

^t^ Independent samples *t*-test/^m^ Mann–Whitney u-test/^X^2^^ Chi-square test (Fisher’s test).

**Table 4:** Diastolic dysfunction analysis before and after kidney transplantation by graft function (GFR < 45 vs. GFR > 45).

		After TX GFR < 45			After TX GFR > 45			*p*	
		Mean ± sd/n%		Median	Mean ± sd/n%		Median		
**TTE**									
**Diastolic Dysfunction**									
Pre-TX	(−)	26	61.9%		73	70.9%		0.293	^X^2^^
	(+)	16	38.1%		30	29.1%			
Post-TX	(−)	17	40.5%		52	50.5%		0.274	^X^2^^
	(+)	25	59.5%		51	49.5%			
Pre-TX/Post-TX Change *p*		0.035	^N^		0.001	^N^			

^X^2^^ Ki-kare test/^N^ McNemar’s test.

**Table 5:** Post-transplant cardiac function analysis by GFR.

		Post-TX GFR < 45				Post-TX GFR > 45				*p*	
		Mean ± sd			Median	Mean ± sd			Median		
**TTE**											
**RV**											
Pre-TX		3.07	±	0.42	3.00	3.06	±	0.41	3.00	0.718	^m^
Post-TX		3.25	±	0.41	3.20	3.23	±	0.45	3.20	0.677	^m^
Pre-TX/Post-TX Change		0.18	±	0.56	0.30	0.17	±	0.49	0.20	0.599	^m^
Change within Group *p*		0.056			^W^	0.000			^W^		
**PAB Max**											
Pre-TX		14.7	±	12.7	9.0	15.8	±	12.0	9.0	0.885	^m^
Post-TX		21.2	±	16.9	9.0	13.2	±	10.3	8.0	0.000	^m^
Pre-TX/Post-TX Change		6.43	±	19.87	0.00	−2.62	±	13.64	0.00	0.006	^m^
Change within Group *p*		0.032			^W^	0.035			^W^		
**EF**											
Pre-TX		60.1	±	8.1	65.0	63.3	±	4.7	65.0	0.004	^m^
Post-TX		61.0	±	9.1	65.0	63.7	±	4.3	65.0	0.043	^m^
Pre-TX/Post-TX Change		0.83	±	9.30	0.00	0.44	±	4.75	0.00	0.070	^m^
Change within Group *p*		0.375			^W^	0.248			^W^		

^m^ Mann–Whitney u-test/^w^ Wilcoxon’s test.

**Table 6:** Analysis of left ventricular hypertrophy (LVH) before and after kidney transplantation.

		Post-TX GFR < 45		Post-TX GFR < 45		*p*	
		n	%	n	%		
**TTE**							
**LVH**							
Pre-TX	(−)	22	52.4%	54	52.4%	0.996	^X^2^^
	(+)	20	47.6%	49	47.6%		
Post-TX	(−)	18	42.9%	55	53.4%	0.250	^X^2^^
	(+)	24	57.1%	48	46.6%		
Pre-TX/Post-TX Change *p*		0.454	^N^	1.000	^N^		

^X^2^^ Ki-kare test/^N^ McNemar’s test.

**Table 7:** Pericardial effusion rates before and after kidney transplantation, analyzed by GFR.

		Post-TX GFR < 45		Post-TX GFR > 45		*p*	
		n	%	n	%		
**TTE**							
**Pericardial Effusion**							
Pre-TX	(−)	38	90.5%	96	93.2%	0.574	^X^2^^
	(+)	4	9.5%	7	6.8%		
Post-TX	(-)	38	90.5%	103	100.0%	0.006	^X^2^^
	(+)	4	9.5%	0	0.0%		
Pre-TX/Post-TX Change *p*		1.000	^N^	0.016	^N^		

^X^2^^ Ki-kare test (Fisher’s test)/^N^ McNemar’s test.

**Table 8:** Echocardiographic changes between pre-emptive KTx patients and non-pre-emptive KTx patients.

	Pre-Emptive KTx				Non-Pre-Emptive KTx				*p*	
	Mean ± sd			Median	Mean ± sd			Median		
**TTE**										
**RV**										
Pre-TX	2.98	±	0.36	3.00	3.12	±	0.43	3.00	0.146	^m^
Post-TX	3.25	±	0.33	3.20	3.22	±	0.49	3.20	0.454	^m^
Pre-TX/Post-TX Change *p*	−0.27	±	0.42	−0.30	−0.10	±	0.55	−0.14	0.056	^m^
	<0.001			^W^	0.072			^W^		
**PAB Max**										
Pre-TX	14.1	±	10.9	9.0	16.4	±	13.0	9.0	0.919	^m^
Post-TX	14.4	±	11.2	9.0	16.2	±	14.2	8.0	0.950	^m^
Pre-TX/Post-TX Change *p*	−0.32	±	15.1	0.00	0.20	±	16.9	0.00	0.631	^m^
	0.940			^W^	0.688			^W^		
**EF**										
Pre-TX	63.3	±	5.4	65.0	61.8	±	6.4	65.0	0.056	^m^
Post-TX	64.0	±	3.6	65.0	62.3	±	7.3	65.0	0.186	^m^
Pre-TX/Post-TX Change *p*	0.61	±	3.14	0.00	0.51	±	7.81	0.00	0.874	^m^
	0.167			^W^	0.302			^W^		
**Septum**										
Pre-TX	1.07	±	0.94	1.00	1.16	±	0.21	1.20	0.001	^m^
Post-TX	1.11	±	0.24	1.10	1.16	±	0.21	1.20	0.041	^m^
Pre-TX/Post-TX Change *p*	−0.04	±	0.21	−0.05	−0.004	±	−0.05	0.00	0.306	^m^
	0.067			^W^	0.821			^W^		
**Posterior**										
Pre-TX	0.98	±	0.17	1.00	1.08	±	0.17	1.10	<0.001	^m^
Post-TX	1.03	±	0.16	1.00	1.09	±	0.21	1.10	0.049	^m^
Pre-TX/Post-TX Change p	−0.05	±	0.18	−0.02	−0.008	±	0.24	0.00	0.186	^m^
	0.049			^W^	0.963			^W^		
**RA**										
Pre-TX	3.20	±	0.32	3.30	3.32	±	0.40	3.30	0.121	^m^
Post-TX	3.33	±	0.30	3.30	3.31	±	0.49	3.30	0.557	^m^
Pre-TX/Post-TX Change p	−0.07	±	0.46	−0.10	−0.008	±	0.57	0.00	0.045	^m^
	0.026			^W^	0.561			^W^		
**LA**										
Pre-TX	3.47	±	0.53	3.43	3.68	±	0.64	3.55	0.041	^m^
Post-TX	3.54	±	0.48	3.50	3.69	±	0.52	3.60	0.082	^m^
Pre-TX/Post-TX Change *p*	0.10	±	0.62	0.10	0.01	±	0.48	0.00	0.492	^m^
	0.175			^W^	0.850			^W^		

^m^ Mann–Whitney u-test/^w^ Wilcoxon’s test. Abbreviations: RV: right ventricle, PAB max: pulmonary artery pressure max, EF: ejection fraction, RA: right atrium, LA: left atrium.

**Table 9:** Echocardiographic changes between pre-emptive KTx patients and non-pre-emptive KTx patients.

			Pre-Emptive KTx		Non-Pre-Emptive KTx		*p*		
			n = 57	39.3%	n = 88	60.7%			
**TTE**									
**Pericardial Effusion**									
Pre-TX		(-)	56	98.2%	78	88.6%	0.033		^X^2^^
		(+)	1	1.8%	10	11.4%			
Post-TX		(-)	55	96.5%	86	97.7%	0.646		^X^2^^
		(+)	2	3.5%	2	2.3%			
Pre-TX/Post-TX Change *p*			1.000	^N^	0.039	^N^			
**LVH**									
Pre-TX	(-)		38	66.7%	38	43.2%		0.006	^X^2^^
	(+)		19	33.3%	50	56.8%			
Post-TX	(-)		33	58.9%	39	44.3%		0.087	^X^2^^
	(+)		23	41.1%	49	55.7%			
Pre-TX/Post-TX Change *p*			0.332	^N^	1.000	^N^			
Pre-TX i/Post-TX Change *p*			0.934	^N^	0.544	^N^			

^X^2^^ Ki-kare test (Fisher’s test);/^N^ McNemar’s test; Abbreviations: KTx: kidney transplantation.

**Table 10:** Echocardiographic changes between pre-emptive KTx patients and non-pre-emptive KTx patients.

		Pre-Emptive KTx			Non-Pre-Emptive KTx			*p*	
		Mean ± sd/n%		Median	Mean ± sd/n%		Median		
**TTE**									
**Diastolic Dysfunction**									
Pre-TX	(−)	46	80.7%		53	60.2%		0.010	^X^2^^
	(+)	11	19.3%		35	39.8%			
Post-TX	(−)	31	54.4%		38	43.2%		0.327	^X^2^^
	(+)	26	45.6%		50	56.8%			
Pre-TX/Post-TX Change *p*		0.001	^N^		0.014	^N^			

^X^2^^ Ki-kare test/^N^ McNemar’s test.

## Data Availability

The datasets used and/or analyzed during the current study are available from the corresponding author on reasonable request.
